# Quality of Life in Epilepsy: A Study of Brazilian Adolescents

**DOI:** 10.1371/journal.pone.0106879

**Published:** 2014-09-05

**Authors:** Nathália F. Siqueira, Fernando L. B. B. Oliveira, Jorge A. Siqueira, Elisabete Abib Pedroso de Souza

**Affiliations:** Neurology Department, State University of Campinas, Campinas, São Paulo, Brazil; University of Modena and Reggio Emilia, Italy

## Abstract

**Objective:**

Our objectives were: to assess the QOL of Brazilian adolescents with epilepsy with a specific QOL assessment tool; to compare the adolescents with epilepsy and healthy adolescents using a generic QOL assessment tool; to correlate the 2 different QOL assessment tools (the generic and the epilepsy-specific); and to correlate QOL scores of the adolescents with epilepsy obtained by both tools with physical, psychological and social variables of the disease.

**Methods:**

Fifty subjects (case group) attending the outpatient clinic of epilepsy of the Clinics Hospital of UNICAMP, Campinas-SP, answered the Brazilian version of the QOL inventory for adolescents with epilepsy - QOLIE-AD-48 and the QOL Questionnaire - WHOQOL – BREF – Portuguese version. Fifty-one subjects (control group) from public schools in Campinas-SP answered only the WHOQOL-BREF.

**Results:**

The results showed that adolescents with epilepsy presented a good score of QOL in both tools; overall scores for both groups with WHOQOL-BREF were good, but a statistically significant difference was seen with regards to the Psychological domain of the WHOQOL-BREF favoring the control group. Significant correlations between QOLIE-AD-48 Total and WHOQOL domains were found. Adolescents that were considered seizure-free (P = 0.001), had good perception of seizure control (P = 0.012) and had not had occurrence of seizures in public places (P = 0.001) presented better QOL scores.

**Conclusions:**

Brazilian adolescents with epilepsy may present good QOL scores when they themselves consider the disease as under control; physical, social and especially psychological variables associated to the disease may play an important role in these results. As a generic QOL tool, WHOQOL-BREF was sufficient to allow for a comparison between chronic disease bearers and healthy adolescents and showed that the gap in QOL between both populations is not as extensive as once was thought, probably due to better support and adaptation to the disease.

## Introduction

Questions related to the subjective well-being began to gain importance when the increase in life expectancy led to an increase in the incidence of chronic diseases, showing the necessity of evaluating the impact of these conditions in the routine of the patients [1]. In this context, Quality of Life (QOL) is considered an important outcome measure for health care [2].

QOL was defined by WHO [3] as “individuals' perception of their position in life in the context of the culture and value systems in which they live and in relation to their goals, expectations, standards and concerns”. QOL is a subjective concept based on the subject's perspective and experiences, and involves physical, psychological, social, and cultural components, all of which contributes to patient's adjustment to his condition and impacts perception in his life [1], [4], [5].

The complexity and extension of the problem that is living with a chronic disease has led many authors to design studies whose objective was to assess the impact of such conditions on the bearer's QOL under the quoted components [5]. The increase in the number of studies in this field demonstrates the importance of the patient's perspective in the evaluation of his own well-being – in different dimensions of the disease process and in the relationship with the environment -, given that biometric measurements are insufficient to assess the impact of the disease [6].

People with chronic disease experience different feelings and behaviors that bring changes in physical capacity, in self-esteem and body image, in relationships with other people and in fulfillment of daily life activities [5], and may be an important stress factor [7].

For quite a while it has been recognized that epilepsy – a chronic disease - affects many aspects of the bearer's life, but only recently systematic evaluations have presented this issue within the theoretical concept that is the background for QOL programs [8].

Epilepsy comes to mean losses in different areas: physical, health, employment, social, family relationships and QOL. In developing countries such as Brazil, epilepsy is a chronic disease that occurs primarily in childhood and adolescence [4].

For adolescents, the fact of knowing they have epilepsy is a strong trigger of psychological and social problems [9]; this leads to significant changes in the individual's life and his family's, not only in physical but also in social and psychological aspects [4]. Epilepsy initiated during childhood and adolescence deserves attention because it may be considered a real risk factor for emotional and behavior problems, leading to an expected decrease in QOL, in later years [10].

The impact of epilepsy extends beyond the seizure experience, and should involve the evaluation of the patient's current reality as well, in which subjective factors are linked to external factors and have a fundamental role in leading to comfort and emotional well-being [6]. To evaluate subjective variables seems to be an important strategy to understand the impact of the disease for the bearer.

Based on this background, our objectives were: to assess the QOL of Brazilian adolescents with epilepsy with a specific QOL assessment tool; to compare the adolescents with epilepsy and healthy adolescents using a generic QOL assessment tool; to correlate the 2 different QOL assessment tools (the generic and the epilepsy-specific); and to correlate QOL scores of the adolescents with epilepsy obtained by both tools with physical (seizure frequency, drug treatment, type of seizure), psychological (perception of seizure control) and social (occurrence of seizure in public places) variables of the disease.

## Material and Methods

### Participants

The study sample consisted of 101 adolescents assessed in the years 2011 and 2012. We assessed 50 subjects attending the outpatient clinic of epilepsy of the Clinics Hospital of UNICAMP, Campinas-SP, Brazil, and 51 subjects without epilepsy selected from public schools in Campinas-SP, Brazil. The inclusion criteria were: age between 10 and 19 years, according to the WHO criteria for adolescents [11], education level between elementary and high school, ability to answer the questions by himself/herself, medical diagnosis of epilepsy for more than 2 years (criteria established in service for epilepsy diagnosis) for the group with epilepsy (case group) and no diagnosis of epilepsy for the group without epilepsy (control group). The exclusion criteria for both groups were: previous brain surgery, use of a concomitant medication with central nervous system effects, or presence of another progressive neurological or psychiatric illness.

### Tools

1 – **Identification card with demographic data** (age, sex, education level, and job), and **epilepsy data** related to physical variables of epilepsy (seizure frequency, type of seizure and drug treatment), psychological variable (perception of seizure control) and social variable (occurrence of seizure in public places). All these data were collected by the researcher; for gathering data regarding epilepsy for the case group (physical variables), the researcher used medical records available at the epilepsy clinic. Regarding seizure frequency, two groups were considered: frequent (had experienced a seizure within the past year) and seizure-free (seizure-free for at least 1 year) – criteria established by the clinic. Type of seizure was classified according to the International League Against Epilepsy classification of epileptic seizures [12]: focal seizures, generalized seizures, focal + generalized seizures. Drug treatment was classified as none, monotherapy (one type of medication) and polytherapy (two or more medications). Data on psychological variable (perception of seizure control) was obtained through patient reports, in which the patients themselves evaluated whether their seizures were controlled or not; these data were subjective and alterations in the intensity and/or frequency of the seizures may influence this variable [6]. Patients were also questioned about the social variable: if they had had occurrence of seizures in public places (yes/no).

2 – **Brazilian version of the QOL inventory for adolescents with epilepsy** **- QOLIE-AD-48** [13] for case group only: QOLIE-AD-48 is a tool developed specifically to assess QOL in adolescents with epilepsy and is comprised of 48 items divided in eight domains: Epilepsy Impact, Memory/Concentration, Attitudes toward Epilepsy, Physical Functioning, Stigma, Social Support, School Behavior, and Health Perceptions. The domain scores were transposed to a scale of 0 to 100: the bigger the score, the better the QOL.

3 – **QOL Questionnaire** **- WHOQOL – BREF – Portuguese version** [14] for both case and control group: it is a tool containing 26 questions that evaluate the QOL in the general population. The WHOQOL-BREF is made of four QOL domains – Physical, Psychological, Social, and Environmental; each domain aims to analyze physical capacity, psychological well-being, social relationships, and the environment where the individual is inserted. The bigger the score, the better the QOL.

### Procedure

Initially the Ethics Committee of UNICAMP approved the research project 176/2005 number. After that, written informed consent was obtained from all subjects and their companions (the one with legal responsibility over the subject), on the day of the outpatient consultation, in an interview to explain the procedures. Subjects were assessed individually at the Psychology Applied to epilepsy outpatient clinic at the Clinics Hospital of UNICAMP. On this first day, the objective was to create a positive bond between the professional and subject (interview/presentation stage). On the second day, one week later, the objective was to apply the tools of research (the assessment stage). The selection of subjects for the case group obeyed the demand of our epilepsy outpatient clinic, according to the criteria for inclusion and exclusion, acceptance of the subject and responsibility to take the research.

The selection of subjects of control group was according to the criteria of inclusion and exclusion, acceptance of the subject and responsibility to take the research. Two public schools in Campinas-SP, Brazil, both of elementary and high-school levels, were chosen by lottery. After the headmaster's authorization, the students were taught about the research and those who had an interest in participating took the written informed consent home to get their parents' signed authorization (interview/presentation stage). All the adolescents of control group were assessed at the individual classroom of their school, when the researcher returned to the school to apply the tools of the research (assessment stage).

### Statistical analysis

Statistical analysis was run in Statistical Package for the Social Science (SPSS) version 18.0 for Windows.

The aims of statistical analysis were to describe the sample profile according to the study variables. Data was comprised of: categorical variables with values of percentage (%), and descriptive statistics (with measurements of position and dispersion - mean, standard deviation, minimum, maximum and median) for continuous variables (scores of scales).

To compare numerical variables between the two groups we used the Mann-Whitney test. In order to compare WHOQOL-BREF score within and between groups, analysis of covariance (ANCOVA) using rank-transformed values to adjust for age and sex was used.

The significance level adopted was 5%, or P<0.05.

## Results

Regarding demographic data, mean years of age of case group was 14.02 (Standard Deviation (SD)  = 1.99) and of control group was 12.63 (SD  = 1.62); this difference was statistically significant (P<0.00001). The majority of adolescents of the case group were male (68%), students (96%), and attending elementary school (72%). Most adolescents of control group were female (58.82%), students (100%), and attending elementary school (90.19%). The difference of gender was statistically significant (P = 0.007).

For specific features of the case group (physical variables), 62% had focal seizures, 56% had frequent seizures and 58% were on monotherapy.

With regards to the psychological variable (perception of seizure control) 84% reported had control perception and with regards to the social variable (occurrence of seizure in public places) 58% reported to have had seizures in public places.

**Table 1** lists the data on QOL assessed by QOLIE-AD-48 for the group with epilepsy (case group).

**Table 1:** Results of QOLIE-AD-48.

	N	Mean	SD	Min	Median	Max
**Epilepsy Impact**	50	75.29	22.12	4.17	82.29	100.00
**Memory/Concentration**	50	66.60	22.59	0.00	70.00	100.00
**Attitudes toward Epilepsy**	50	35.00	24.05	0.00	37.50	81.25
**Physical Functioning**	50	85.20	23.18	0.00	95.00	100.00
**Stigma**	50	64.69	23.73	0.00	64.14	100.00
**Social Support**	50	86.12	19.20	25.00	96.87	100.00
**School Behavior**	50	84.37	19.77	0.00	87.50	100.00
**Health Perceptions**	50	67.16	15.74	25.00	66.66	100.00
**Total**	50	68.73	15.09	21.35	72.08	96.02

SD: standard deviation; Min.: minimum; Max.: maximum; N: number of subjects.

The adolescents with epilepsy showed good QOL scores except in the Attitudes toward Epilepsy domain.

**Table 2** lists the data on QOL assessed by WHOQOL-BREF, in both groups (case and control groups). Of the initial 50 subjects in the case group, only 28 subjects took the WHOQOL-BREF.

**Table 2:** Results of WHOQOL-BREF in both groups.

Domains	N	Mean	SD	Min	Median	Max	P Value*
**Case Group**							
Physical	28	72.57	12.14	50.00	71.42	100.0	
Psychological	28	64.43	13.10	41.67	64.58	91.67	
Social	28	73.21	17.32	25.00	75.00	100.0	
Environmental	28	63.72	13.56	37.50	65.62	87.50	
**Control Group**							
Physical	51	76.47	13.70	46.43	75.00	100.0	0.428
Psychological	51	72.38	16.60	16.67	75.00	91.67	0.016
Social	51	75.16	19.40	16.67	75.00	100.0	0.184
Environmental	51	66.42	15.49	0.00	65.62	90.63	0.287

SD: standard deviation; Min.: minimum; Max.: maximum; N: number of subjects. Correlations are in bold style.

*P value of the ANCOVA rank-transformed values covariates age and sex. * Significant at p< .05.

Overall results were considered good for both groups. A statistically significant result was found in the Psychological domain of the WHOQOL (P = 0.016) favoring the control group.

**Table 3** lists the correlations between seizure frequency and QOLIE-AD-48 and WHOQOL-BREF.

**Table 3:** Correlations between seizure frequency and QOLIE-AD-48 and WHOQOL-BREF.

Seizure Frequency	N	Mean	SD	Min	Median	Max	P Value*
**WHOQOL Physical**							
Frequent	16	70.75	12.98	50.00	71.42	100.00	
Seizure free	12	75.00	10.98	57.14	71.42	96.43	0.424
**WHOQOL Psychological**							
Frequent	16	60.15	13.52	41.67	58.33	87.50	
Seizure free	12	70.13	10.48	54.17	70.83	91.67	0.047
**WHOQOL Social**							
Frequent	16	68.22	19.05	25.00	75.00	100.00	
Seizure free	12	79.86	12.54	58.33	83.33	100.00	0.064
**WHOQOL Environmental**							
Frequent	16	63.28	16.27	37.50	64.06	87.50	
Seizure free	12	64.32	9.46	50.00	65.62	81.25	0.963
**QOLIE-AD-48 Total**							
Frequent	28	63.31	14.45	21.35	65.54	85.75	
Seizure free	22	75.63	13.20	41.80	78.48	96.02	0.001

* P value referring to the Mann-Whitney for comparison of the variables frequent x seizure-free. Correlations are in bold style.

The results showed significant correlations between seizure frequency and WHOQOL-Psychological domain and QOLIE-AD-48-Total.

**Table 4** lists the correlations between perception of seizure control and QOLIE-AD-48 and WHOQOL-BREF.

**Table 4:** Correlations between perception of seizure control and QOLIE-AD-48 and WHOQOL-BREF.

Perception of seizure control	N	Mean	SD	Min	Median	Max	P Value*
**WHOQOL Physical**							
Controlled	24	72.76	12.76	50.00	71.42	100.00	
Not controlled	04	71.42	8.74	60.71	71.42	82.14	0.816
**WHOQOL Psychological**							
Controlled	24	64.06	13.61	41.67	64.58	91.67	
Not controlled	04	66.66	10.75	54.17	66.66	79.17	0.668
**WHOQOL Social**							
Controlled	24	72.91	18.59	25.00	75.00		
Not controlled	04	75.00	6.80	66.67	75.00		0.893
**WHOQOL Environmental**							
Controlled	24	63.02	12.79	37.50	64.06		
Not controlled	04	67.96	19.32	40.63	73.43		0.410
**QOLIE-AD-48 Total**							
Controlled	42	71.38	13.08	41.80	74.55		
Not controlled	08	54.82	18.18	21.35	58.87		0.012

* P value referring to the Mann-Whitney for comparison of the variables controlled x not controlled. Correlations are in bold style.

The results showed significant correlations between perception of seizure control and QOLIE-AD-48 Total.

**Table 5** lists the correlations between occurrence of seizure in public places and QOLIE-AD-48 and WHOQOL-BREF.

**Table 5:** Correlations between occurrence in public places and QOLIE-AD-48 and WHOQOL-BREF.

Occurrence in public places	N	Mean	SD	Min	Median	Max	P Value*
**WHOQOL Physical**							
Yes	16	70.08	13.29	50.00	71.42	100.00	
No	12	75.89	10.00	64.29	73.21	96.43	0.204
**WHOQOL Psychological**							
Yes	16	62.50	12.63	41.67	60.41	87.50	
No	12	67.01	13.81	41.67	68.75	91.67	0.338
**WHOQOL Social**							
Yes	16	72.91	15.95	33.33	75.00	100.00	
No	12	73.61	19.73	25.00	75.00	100.00	0.704
**WHOQOL Environmental**							
Yes	16	64.25	15.43	37.50	67.18	87.50	
No	12	63.02	11.21	46.88	62.50	81.25	0.709
**QOLIE-AD-48 Total**							
Yes	29	63.61	14.12	21.35	65.04	88.69	
No	21	75.80	13.73	41.80	79.56	96.02	0.001

* P-value referring to the Mann-Whitney for comparison of the variables occurrence of seizures in public places x absence of seizure in public places. Correlations are in bold style.

The results showed significant correlations between occurrence of seizure in public places and QOLIE-AD-48-Total.

For the other physical variables associated with epilepsy, the results showed no significant correlations between drug treatment and WHOQOL domains (Physical P = 0.878; Psychological P = 0.929; Social P = 0.693; Environmental P = 0.673) and QOLIE-AD-48-Total (P = 0.079). The correlations were also not significant between type of seizure and WHOQOL domains (Physical P = 0.517; Psychological P = 0.496; Social P = 0.954; Environmental P = 0.614) and QOLIE-AD-48 (P = 0.294).

The correlations between both QOLIE-AD-48 and WHOQOL-BREF tools are presented in **Table 6** **.**

**Table 6:** Correlations between QOLIE-AD-48 and WHOQOL-BREF.

QOLIE-AD-48	WHOQOL Physical	WHOQOL Psychological	WHOQOL Social	WHOQOL Environmental
**Total**	*r = 0.184	**r = 0.549**	r = 0.253	r = −0.121
	(P = 0.349)	(P = 0.002)	(P = 0.195)	(P = 0.540)
**Epilepsy Impact**	r = −0.126	r = 0.269	r = −0.043	r = −0.270
	(P = 0.522)	(P = 0.166)	(P = 0.829)	(P = 0.164)
**Memory/Concentration**	**r = 0.383**	**r = 0.608**	r = 0.158	r = 0.037
	(P = 0.045)	(P = 0.001)	(P = 0.421)	(P = 0.853)
**Attitudes toward Epilepsy**	r = 0.085	r = 0.243	r = 0.025	r = −0.054
	(P = 0.666)	(P = 0.213)	(P = 0.900)	(P = 0.784)
**Physical Functioning**	r = −0.045	**r = 0.393**	r = 0.059	r = 0.081
	(P = 0.822)	(P = 0.039)	(P = 0.767)	(P = 0.681)
**Stigma**	r = 0.128	**r = 0.476**	r = 0.230	r = 0.022
	(P = 0.517)	(P = 0.010)	(P = 0.240)	(P = 0.913)
**Social Support**	r = 0.135	r = 0.164	r = 0.226	r = 0.368
	(P = 0.492)	(P = 0.405)	(P = 0.247)	(P = 0.054)
**School Behavior**	**r = 0.459**	r = 0.356	r = 0.136	r = 0.287
	(P = 0.014)	(P = 0.063)	(P = 0.491)	(P = 0.139)
**Health Perceptions**	**r = 0.471**	**r = 0.417**	r = 0.359	**r = 0.398**
	(P = 0.011)	(P = 0.027)	(P = 0.060)	(P = 0.036)

Notes: * r =  Spearman correlation coefficient; P =  P value. Correlations are in bold style.

The results show significant correlations between QOLIE-AD-48-Total and WHOQOL–Psychological domain; between QOLIE-AD-48-Memory/Concentration and WHOQOL–Physical and Psychological domains as well as QOLIE-AD-48-Physical Functioning and WHOQOL-Psychological domain; QOLIE-AD-48-Stigma and WHOQOL-Psychological domain; QOLIE-AD-48-School Behavior and WHOQOL-Physical domain; and QOLIE-AD-48-Health Perceptions and WHOQOL-Physical, Psychological and Environmental domains.

## Discussion

It is now well recognized that epilepsy has a major impact on the patient's QOL. Earlier studies focusing on patients with epilepsy have shown that these patients have had their QOL negatively affected by the disease [15], [16], [17].

Our present study showed that Brazilian adolescents with epilepsy present a relatively good QOL score (68.73±15.09) when assessed by a population-specific tool (QOLIE-AD-48). QOL measurement tools consider the bigger the score, the better the QOL; we considered an arbitrary cutoff value of more than 60 points as good QOL. The scores were good in the assessed domains, with the exception of Attitudes toward Epilepsy domain (35.00±24.05). We found a few studies that corroborate with our results.

The study of Wu et al [18] identified a high level of QOL of adolescents with epilepsy in China as well. QOL was measured using the Chinese version of QOLIE-AD-48. The mean total score of QOLIE-AD-48 of epileptic subjects was 65.6±14.1, which was considered high by the authors.

In the study of Barbosa et al [13], that had as objectives to translate and adapt the Quality of Life in Epilepsy Inventory for Adolescents (QOLIE-AD-48) into Brazilian Portuguese, the study subjects (Brazilian adolescents) got a mean total score of 69.91±11.44 except in the Attitudes toward Epilepsy domain (39.31±19.99), results similar to ours.

Using the same QOLIE-AD-48, Stevanovic [19] found even better results. The mean QOL total scores were 83.9 for boys and 83.06 for girls in Serbia. The highest scores were observed in the Physical Functioning and School Behavior domains; the lowest in the Attitude toward Epilepsy domain, the same results we found in our study.

The sum of our results and the ones quoted points out to a good QOL score measured by QOLIE-AD-48 in this population (even though from different countries). It is certain that adaptation to the disease and perception of quality of life play important roles in these studies.

When comparing adolescents with epilepsy and healthy ones using a generic tool – WHOQOL-BREF -, QOL scores were generally similar to both groups, and considered good as well. It was possible to identify a statistically significant difference with regards to the Psychological domain of the WHOQOL-BREF (P = 0.006) favoring the control group. Such results may suggest that adolescents in the case group possess less control over psychological variables when compared to the control group, certainly due to the disease the case group bears.

Our literature review did not identify studies using the WHOQOL-BREF as a tool for assessing QOL of adolescents with epilepsy; only studies with adults were found. In the study of Liou et al [20], adult patients with epilepsy had poorer QOL than the healthy population in Physical, Psychological and Social domains of the WHOQOL-BREF but not in the Environmental domain. Huang et al [21] evaluated adult patients with epilepsy and the results revealed that scores on two domains of the WHOQOL-BREF (Physical and Psychological domains) were significantly lower in the epilepsy group compared with the control group. While adults with epilepsy presented poor overall scores when compared to healthy ones in more than one domain, our study with adolescents with epilepsy showed good overall scores when compared to healthy ones – which presupposes that these adolescents with epilepsy may have good disease support (more understanding of the disease, proper adaptation, etc.), leading to a QOL near the expected for healthy adolescents; but the Psychological domain still reflected the presence of the disease (statistically significant difference).

When we correlated the physical, psychological and social variables of epilepsy with both QOL measurement tools, we found important results for our discussion. We hypothesized that physical, psychological and social disease-specific variables such as seizure frequency, perception of seizure control and occurrence of seizures in public places may be related to the good QOL scores obtained. The correlations showed positive results – adolescents that were considered seizure-free, had good perception of seizure control and had not had occurrence of seizures in public places presented better QOL scores in the QOLIE-AD-48. We also found a positive correlation between seizure-free and the WHOQOL-Psychological domain.

All three variables work together to deliver a notion of controlled disease and good QOL. Seizure frequency and absence of seizures in public places are variables that have, in their core, the pharmacological treatment component – if the patient is adequately treated with pharmacological interventions, it is assumed that seizures will be controlled. But the perception of seizure control has a psychological component (patient's own perspective) in its core, and may have the strongest influence over the perceived QOL reported by the adolescents. Our results might be explained by the good perception of seizure control (84% of subjects studied) and good adjustment to epilepsy presented by the subjects. Other studies also found out that patients with well-controlled epilepsy report a high level of psychological functioning and self-esteem and a low level of distress and anxiety, as well as better adjustment to the disease. These studies also showed that good perception of seizure control seems to facilitate social contacts and a normal life [22], [23].

Epilepsy patients have the perception of their illness through their seizures [24]. Souza and Salgado [6] draw attention to the perception of seizure control as an important factor in the control of the disease, even if, from the medical perspective, the disease is not considered controlled. The patient may have frequent seizures, but these decreased in the current period as he may deem it under control. According to the authors, the perception of seizure control seems to be an important factor to mitigate the impact of potentially stressful experiences. Studies of Gertrudis et al [25] and Cancian [26] also point out that the way the individual experiences his seizures has no direct relationship with the severity of seizures, but with adaptation strategies that individuals adopt and the ways they define their clinical, social and personal realities. The use of more positive coping strategies can change the meaning of the seizures, making the situation more acceptable.

QOL is a multidimensional concept that evaluates many aspects of human condition. This complex interaction between internal and external factors, in a chronic disease, suffers modifications. In a chronic disease like epilepsy, the subject may be controlled by different variables, contingencies that are linked to the manner with which individuals perceive themselves and perceive their relationship with the environment. Our results suggest that physical (seizure-free), social (absence of occurrence of seizures in public places) and especially psychological (good perception of seizure control) variables seem to favor a good QOL perception.

Our study also evaluated the correlations between domains from the 2 different tools, and positive correlations were found, especially between QOLIE-AD-48 domains (Total, Memory/Concentration, Physical Functioning, Stigma and Health Perceptions) and the WHOQOL Psychological domain, and between QOLIE-AD-48 domains (Memory/Concentration, School Behavior and Health Perceptions) and the WHOQOL Physical domain. Considering that correlations were found for the Physical and Psychological domains of WHOQOL-BREF with QOLIE-AD-48 domains, we may infer that both instruments are promising tools for assessing QOL in clinical evaluation of and research on adolescents with epilepsy. For the purpose of comparing the gap between QOL of populations with and without a chronic disease, WHOQOL-BREF may be used, but with a careful analysis of the results.

It was expected that the tools would show more correlations considering that they assess the same construct. QOLIE-AD-48 is a specific tool for adolescents with epilepsy and has a focus on inherent difficulties faced by this population. In a sense, QOLIE-AD-48 considers the disease as the sole driver of QOL. WHOQOL-BREF is more general, not age-specific and with a small number of questions.

The generic tools may be used in both healthy populations and individuals affected by a specific condition, focusing on their experience and subjective perceptions. The specific tools are more related to the daily QOL of the bearer when it comes to disease experience, worsening states or medical interventions, the purpose being the identification of the associated problems. They focus on symptoms, function and incapacities; they provide a more accurate observation regarding the implication of different treatments, allowing a comparison of the impact of alternative treatments and evaluation of the proposed interventions [27]. For a patient who perceives his disease as controlled, a disease-specific assessment tool will probably show good QOL scores, while a generic one, whose focus is not in the disease, may be able to “identify” the disease within the general QOL scores. Adaptation to the disease and increased perception of QOL due to such adaptation may be sufficient to allow for good scores in the QOLIE-AD-48, but may not be enough to positively affect the Psychological domain of the WHOQOL-BREF.

QOL measurement tools do not stratify QOL in classification scores such as low, medium or high, considering only that the bigger the score, the better the QOL. However, each author arbitrarily – using his own particular criteria - classifies the scores obtained in his research in order to compare results with other studies. We feel it is necessary that a classification with established thresholds be formalized to allow comparison of different populations.

The study had some limitations. The number of subjects was small and the sample was not homogenous because of the study criteria. However, it adds up to the current literature bringing up the evidence that, even though epilepsy is an important influence factor in QOL (it still affects the Psychological domain of the WHOQOL-BREF tool), support, adaptation to and well-controlled variables (physical, psychological and social) of the disease significantly affect QOL of adolescents with epilepsy (good correlations, good overall WHOQOL-BREF and QOLIE-AD-48 scores), elevating it to levels within those of healthy adolescents.

## Conclusions

Brazilian adolescents with epilepsy may present good QOL scores when they themselves consider the disease as under control; physical, social and especially psychological variables associated to the disease may play an important role in these results. As a generic QOL tool, WHOQOL-BREF was sufficient to allow for a comparison between chronic disease bearers and healthy adolescents and showed that the gap in QOL between both populations is not as extensive as once was thought, probably due to better support and adaptation to the disease.

Our knowledge of the behavior of the patient with epilepsy and the questions related to well-being are still incomplete. There remain many investigations related to the subjective process involved in the disease, mainly with adolescents with epilepsy, since adolescence is a transitional stage of physical and mental human development and may have a great influence in the life stages that follow.

To look for other assessment parameters aside from the physical one amplifies the possibilities of analyzing the adolescent within a context and, when identifying difficulties, be able to address them.

To investigate, assess, and treat the problems and difficulties faced by adolescents with epilepsy, as well as understand how this population is experiencing their disease state, are fundamental strategies to allow them to develop and build a healthy professional and social life. To provide behavior changes by offering support to enhance the adaptation of individuals with epilepsy (such as psychoeducation, psychotherapy, social support, and appropriate pharmacological treatment) is essential in prevention and intervention programs for this population.
